# Benefit of multi-hole self-expandable metal stent placement for hilar biliary bleeding: effective hemostasis without biliary branch obstruction

**DOI:** 10.1055/a-2842-0708

**Published:** 2026-04-20

**Authors:** Takehiko Koga, Keisuke Matsumoto, Yusuke Ishida, Yasuharu Shimoji, Mitsuhiro Kiya, Naoaki Tsuchiya, Fumihito Hirai

**Affiliations:** 1 38068 Department of Gastroenterology and Medicine, Fukuoka University Faculty of Medicine, Fukuoka, Japan

**Fig. 1:**
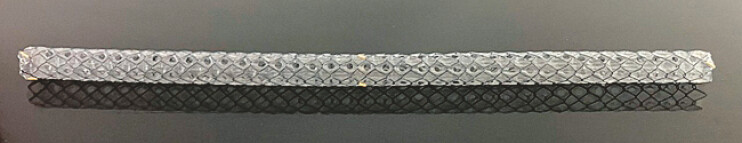
A multi-hole self-expandable metal stent. The stent consists of a hook-and-cross type nitinol wire mesh covered with a silicone membrane. Multiple small holes are incorporated on the silicone covering.

Endoscopic retrograde cholangiopancreatography (ERCP) is an established approach for managing hemobilia associated with hepatobiliary malignancies, and hemostasis using a covered self-expandable metal stent (SEMS) has been reported 1 2 3 . However, in the case of hilar biliary bleeding, this approach may occlude adjacent biliary branches. Herein, we report a novel technique using a multi-hole SEMS (MHSEMS, Fig. 1 , 4 5 ) that achieves hemostasis while preserving biliary branches ( Video 1 ).

Endoscopic hemostasis using a multi-hole self-expandable metal stent.Video 1

**Fig. 2:**
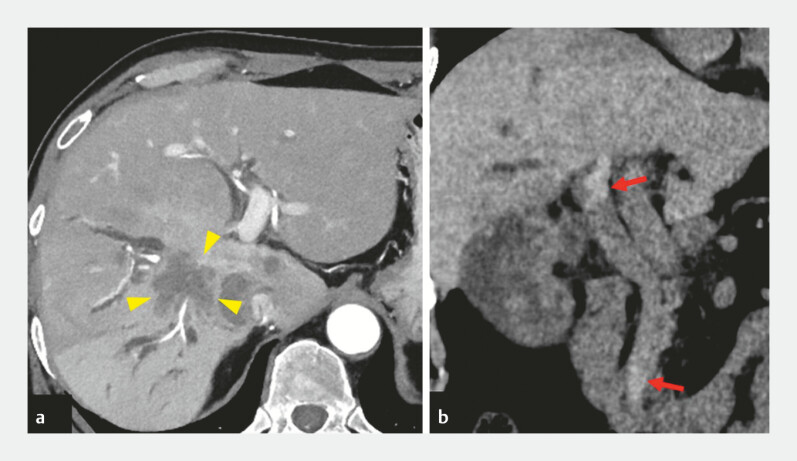
Computed tomographic images. **a** An axial image showing a metastatic tumor in the right hepatic lobe invading the bile ducts (arrowheads). **b** A coronal image demonstrating high-density nodules within the common bile duct (arrows), suggestive of hemobilia.

**Fig. 3:**
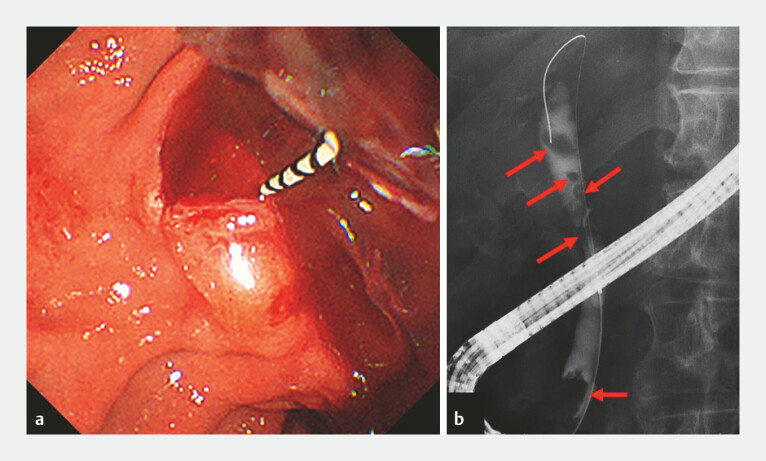
Endoscopic and fluoroscopic findings. **a** An endoscopic image showing active bleeding from the duodenal papilla. **b** Cholangiogram showing filling defects within the bile duct (arrows).

**Fig. 4:**
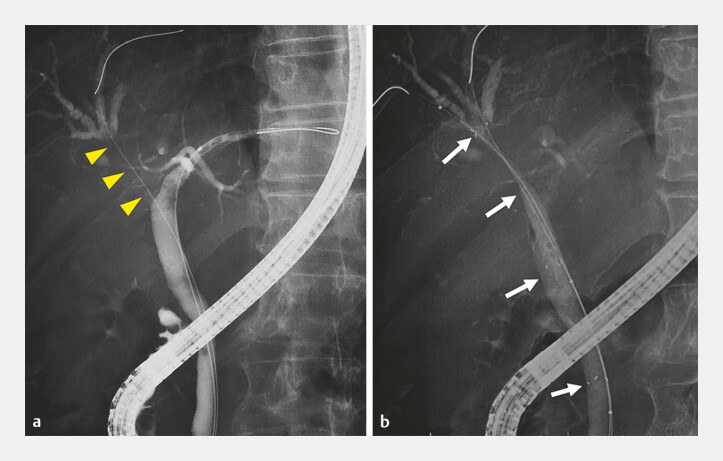
Cholangiography before and after stent placement. **a** A cholangiogram showing the obstruction of the right hepatic duct due to tumor invasion (arrowheads). **b** A cholangiogram after the deployment of the multi-hole self-expandable metal stent from the B8 to the common bile duct (arrows).

**Fig. 5:**
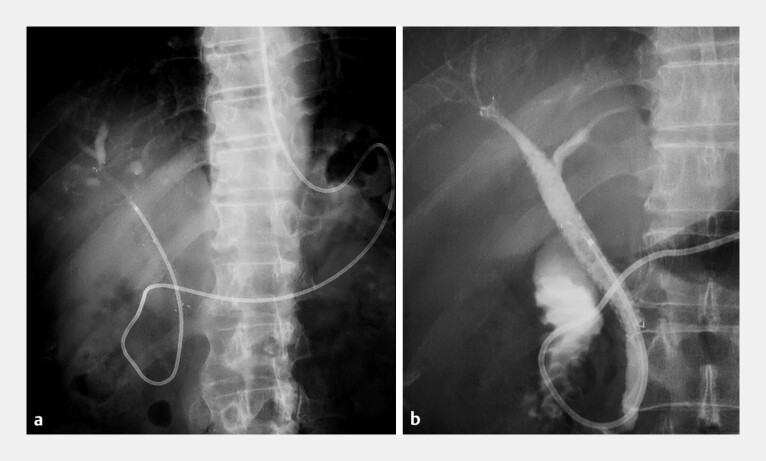
Fluoroscopic images after the procedure. **a** A fluoroscopic image obtained immediately after the procedure, showing the stent positioned from segment B8 to the common bile duct with an endoscopic nasobiliary drainage (ENBD) tube inserted through it. **b** A follow-up cholangiogram performed through the ENBD tube demonstrating contrast flow into the left hepatic duct through the multi-hole stent and no filling defects within the bile duct.

A 68-year-old man with gallbladder cancer and multiple liver metastases presented with jaundice and anemia. Computed tomography revealed high-density nodules within the bile duct, suggesting hemobilia due to bile duct invasion by a metastatic liver lesion ( Fig. 2 ). ERCP revealed active bleeding from the duodenal papilla and filling defects on cholangiography ( Fig. 3 ). The right hepatic duct was obstructed immediately above the hilum by metastatic invasion, whereas the left hepatic duct remained patent. After guidewire placement into the B8 bile duct, the MHSEMS (8 mm × 10 cm, HANAROSTENT Multi-Hole Benefit, M.I.Tech Co., Seoul, Korea) was deployed from B8 to the common bile duct across the left hepatic duct ( Fig. 4 ). An endoscopic nasobiliary drainage (ENBD) tube was placed within the MHSEMS for monitoring. Post-procedural ENBD drainage was non-bloody, and follow-up cholangiography via the ENBD showed no filling defects and smooth contrast flow into the left hepatic duct through the MHSEMS ( Fig. 5 ). The patient’s condition improved, and chemotherapy was resumed.

Compared with the conventional covered SEMS, the MHSEMS has a smaller silicone-membrane contact area at the bleeding site but a lower risk of occluding the biliary branches. Although the safety of MHSEMS placement in the hilar region remains uncertain due to potential risks such as segmental cholangitis and early obstruction, the MHSEMS may provide an optimal balance between hemostasis and preservation of biliary drainage in selected cases of hilar biliary bleeding.

Endoscopy_UCTN_Code_TTT_1AR_2AZ
